# Improvement of *Mycobacterium tuberculosis* detection by Xpert MTB/RIF Ultra: A head-to-head comparison on Xpert-negative samples

**DOI:** 10.1371/journal.pone.0201934

**Published:** 2018-08-13

**Authors:** Francesco Bisognin, Giulia Lombardi, Donatella Lombardo, Maria Carla Re, Paola Dal Monte

**Affiliations:** Department of Experimental, Diagnostic and Specialty Medicine - Unit of Microbiology, Alma Mater Studiorum University of Bologna - S. Orsola-Malpighi University Hospital, Bologna, Italy; Indian Institute of Technology Delhi, INDIA

## Abstract

**Background:**

The new Xpert MTB/RIF Ultra assay (Ultra, Cepheid, Sunnyvale, USA) is a cartridge-based automated diagnostic test that can simultaneously identify *Mycobacterium tuberculosis complex* (MTB) and resistance to Rifampicin (RIF). With respect to the previous version Xpert MTB/RIF assay (Xpert), IS6110/IS1081 repetitive elements probes have been added allowing the detection of lower MTB load, defined by the new semi-quantitative category “trace” with indeterminate RIF resistance. The aim of this study was to evaluate performance of the new version Ultra on Xpert-negative, but TB culture-positive clinical samples.

**Methods:**

The de-identified frozen samples (-20 °C) collected over a 4-year period (February 2014-October 2017), which had previously resulted smear-negative, Xpert-negative but MTB culture-positive, were analyzed with Ultra. The de-frosted samples were loaded into the cartridge using the same process as the previous version, according to manufacturer’s instruction.

**Results:**

During the study period 382 MTB culture-positive samples were archived: 314 resulted Xpert-positive and 68 Xpert-negative. Thirty-one of the 68 Xpert-negative samples resulted positive with Ultra, with an overall improvement in MTB detection of 45.6%. Out of 36 Xpert-negative respiratory samples, 18 resulted Ultra-positive with the following semi-quantitative loads: “low”(n = 1), “very low”(n = 11), “trace”(n = 6), with an improvement in MTB detection of 50%. The best performance was achieved on bronchoalveolar lavage specimens (53.8%). Out of 32 Xpert-negative non-respiratory samples, 13 resulted Ultra-positive with the following semi-quantitative loads: “very low”(n = 7), “trace”(n = 6), with an improvement in MTB detection of 40.6%. The best performance was achieved on biopsies (55.6%) and lymph nodes (50%). The new category “trace” detected 12 out of the 31 Ultra-positive MTB samples; in the remaining 19 samples RIF susceptibility was determined with 100% concordance with the phenotypic susceptibility test. The mean time to positivity of samples found negative by Ultra was significantly longer in comparison to positive samples in liquid culture.

**Conclusions:**

Our results are consistent with the few studies published so far and confirm the better performance of Ultra compared to the previous version in both respiratory and non-respiratory smear-negative samples, with an overall improvement of 45.6%.

## Introduction

Tuberculosis (TB) currently represents a major global health problem, both in developing countries and in Europe, in correlation with migratory flows from high incidence countries. According to the latest WHO estimates, the global incidence of TB in 2016 was 10.4 million new cases and TB accounted for about 1.7 million deaths worldwide [1].

Main concerns about microbiological diagnosis of TB are related to the detection limit of smear microscopy, long time to culture-confirmation and variable sensitivity of molecular tests, particularly for diagnosing smear-negative TB.

The Xpert MTB/RIF assay (Xpert, Cepheid, USA) is a cartridge-based automated diagnostic test that can simultaneously identify *Mycobacterium tuberculosis complex* (MTB) and resistance to Rifampicin (RIF) [2]. Despite substantially greater sensitivity for MTB detection compared with smear microscopy, its sensitivity is inadequate in smear-negative tuberculosis (TB) patients, including those with extra-pulmonary forms [3–5].

The new version Xpert MTB/RIF Ultra (Ultra) has recently been introduced onto the market: the assay sensitivity has been improved by: i) targeting the multicopy IS6110 and IS1081 genes for the detection of MTB, ii) more rapid thermal cycling with fully nested nucleic acid amplification, iii) improved fluidics and enzymes, iv) a larger DNA reaction chamber than the Xpert. As a result, the assay can even detect “traces” of MTB DNA (a new semi-quantitative category with indeterminate RIF resistance) which were undetectable with the previous version of Xpert which targeted only the Rifampicin Resistance-Determining Region (RRDR) of the MTB *rpoB* gene [6]. The limit of detection of Ultra is 15.6 bacterial colony-forming units per ml compared to 114 colony-forming units per ml of Xpert.

So far, few studies have been published about Ultra performance. In particular, comparative analysis of Xpert and Ultra has only been performed in 3 multicentre validation and diagnostic accuracy studies [6–8] that demonstrated the better performance of Ultra with respect to Xpert.

The aim of this study was a head-to-head comparison between Ultra and Xpert on respiratory and non-respiratory samples previously found Xpert-negative MTB culture-positive in order to assess the improvement of smear-negative TB detection.

## Materials and methods

### Study design

This is a retrospective monocentric study performed at the Unit of Microbiology of the S. Orsola-Malpighi University Hospital, referral centre for the diagnosis of TB for the metropolitan area of Bologna (Italy), which processes approximately 5000 MTB cultures and performs about 1000 Xpert analyses per year. The de-identified frozen samples (-20 °C) collected over a 4-year period (February 2014-October 2017), which had previously resulted smear-negative, Xpert-negative but MTB culture-positive, were analyzed with Ultra to assess increase in sensitivity of MTB detection. This study was approved by the Ethics Committee of S. Orsola-Malpighi University Hospital (Protocol code n. 315/2017/O/Tess).

### Sample processing

All specimens were stained for acid-fast microscopic examination using Ziehl-Neelsen stain, before sample concentration. MTB isolation was performed as described in detail previously [9]. Briefly, samples were decontaminated with N-Acetyl-L-Cysteine-Sodium Hydroxide (NALC-NaOH) method and resuspended in 2 ml Phosphate Buffer Saline (PBS): 500 μl were inoculated onto 2 solid slant media (Lowenstein-Jensen; Heipha Diagnostika Biotest, Germany) and 500 μl into liquid culture (MGIT 960; Becton Dickinson, USA). Solid and liquid cultures were considered negative after 42 days of incubation without isolation of any Mycobacteria. Positive cultures were identified as MTB by MGIT TBc Identification Test (Becton Dickinson). Susceptibility of MTB isolates to first-line drugs (Isoniazid, Rifampicin, Ethambutol, Pyrazinamide) was tested by the “gold standard” automatic MGIT 960 system.

“Time to positivity” (TTP) was defined as the number of days from MGIT inoculation to the positive culture result using Epicenter software (Becton Dickinson). An aliquot of 500 μl was tested with Xpert MTB/RIF assay (Xpert, Cepheid, USA) according to manufacturer’s instruction. An aliquot of 500 μl was stored at -20 °C for further use; when culture results were available, only MTB-positive aliquots were archived at -20 °C while the MTB-negative aliquots were discarded.

### Xpert Ultra procedure

Samples found Xpert-negative MTB culture-positive were de-frosted: 500 μl were mixed in a 1:3 ratio with Sample Reagent for 15 minutes at room temperature and poured into a single-use disposable cartridge of the GeneXpert module according to manufacturer’s instruction [10]. The system automatically interpreted the fluorescent signals into the following categories: invalid (if PCR inhibitors were detected with amplification failure), negative or positive. Positive results were divided into 5 categories depending on bacterial load: the relationship between the *rpoB* Ct value and input CFU allows samples to be classified as “high,” “medium, “low,” and “very low” and to define strains as susceptible or resistant to Rifampicin depending on the detection of mutations in the *rpoB* gene, while the “trace” category identifies the paucibacillary samples which are IS6110/IS1081 positive but *rpoB* negative, with indeterminate Rifampicin susceptibility.

### Statistical analysis

Comparison between Ultra-positive and Ultra-negative samples for mean time to culture positivity (TTP) and mean time of storage was performed by Student’s t-test.

Chi square test was used to assess the correlation between the proportion of Ultra-positive results in samples stored more and less than 500 days.

Correlation between IS1081/IS6110 and *rpoB* probes Ct values and TTP or time of storage was performed by regression analysis (Pearson).

Statistical significance was set at p<0.05. Statistical analysis was performed using the STATA SE 14.1 College station (USA).

## Results

During the study period 382 MTB culture-positive samples were archived: 314 resulted Xpert-positive and 68 Xpert-negative. Thirty-six of the Xpert-negative samples were respiratory (26 bronchoalveolar lavages and 10 sputa) and 32 non-respiratory (10 lymph nodes, 9 biopsies, 8 cavitary fluids, 3 urine samples and 2 gastric aspirates).

Thirty-one of the Xpert-negative MTB culture positive samples resulted positive with Ultra, with an overall improvement in MTB detection of 45.6% (31/68). No invalid or error results were obtained.

As reported in Table 1, eighteen of the 36 Xpert-negative respiratory samples tested Ultra-positive with the following semi-quantitative loads: “low” (n = 1), “very low” (n = 11), “trace” (n = 6), while 18 samples remained negative. Therefore Ultra improved MTB detection in smear-negative respiratory samples by 50% (18/36), with the best performance on bronchoalveolar lavages (14/26, 53.8%).

**Table 1:** Mean “time to positivity” and Ultra results of 68 Xpert-negative culture-positive samples according to type of specimen and semi-quantitative load.

Mean TTP^#^ days±sd	Ultra results	TOTAL n = 68	RESPIRATORY n = 36		NON-RESPIRATORY n = 32				
			BAL* n = 26	Sputum n = 10	Lymphnode n = 10	Biopsy n = 9	Cavitary fluid n = 8	Gastric aspirate n = 2	Urine n = 3
**16.4 ± 3.4**	**Positive**	**31**	**14**	**4**	**5**	**5**	**2**	**1**	**0**
17.5	Low	1	0	1	0	0	0	0	0
15.8 ± 3.2	Very low	18	10	1	3	2	1	1	0
17.0 ± 3.7	Trace	12	4	2	2	3	1	0	0
**24.1 ± 9.1**	**Negative**	**37**	**12**	**6**	**5**	**4**	**6**	**1**	**3**

^**#**^Time to positivity: number of days from time of MGIT inoculation to the positive culture result

* Bronchoalveolar lavage

Thirteen of the 32 Xpert-negative non-respiratory samples tested Ultra-positive with the following semi-quantitative loads: “very low” (n = 7), “trace” (n = 6), while 19 samples remained negative, with an improvement in MTB detection of 40.6% (13/32) in smear-negative non-respiratory samples. The best performance was achieved on biopsies (5/9, 55.6%) and lymph nodes (5/10, 50%) (Table 1).

“Time to positivity” (TTP) in MGIT culture was available for 64 samples, while 4 samples were positive only in solid culture (3 resulted Ultra-negative and 1 “very low”). The mean TTP value of samples found positive by Ultra assay was significantly shorter (16.4 ± 3.4 days) than those found negative (24.1 ± 9.1 days, p<0.001, Table 1).

The decontaminated samples were stored at -20°C for 49 to 1389 days (mean time 520.4 ± 357.3 days) before Ultra analysis. Ultra-positive samples had a mean time of storage of 467.5 ± 344.0 days, not significantly different from Ultra-negative ones (564.6 ± 374.4 days, p = 0.22). In samples stored for less than 500 days Ultra resulted positive in 52.8% against 37.5% in samples stored for more than 500 days (p = 0.31).

In Table 2, IS1081 and IS6110 Ct values of the 31 Ultra-positive samples according to specimen types and *rpoB*1-*rpoB*4 Ct values for samples resulting “low” or “very low” are reported. No correlation between IS1081/IS6110 and *rpoB* probes Ct values and TTP or time of storage was observed by regression analysis (data not shown).

**Table 2:** Description of Xpert-negative Ultra-positive samples (n = 31) according to specimen types, storage time, “time to positivity”, semi-quantitative load and Ct results.

SAMPLE	Storage time days	TTP^#^ days	ULTRA LOAD	Ct IS1081 and IS6110	Ct *rpo*B1	Ct *rpo*B2	Ct *rpo*B3	Ct *rpo*B4
**Sputum**	939	17.5	Low	20.1	28.5	28.3	28.6	36.1
BAL*	1304	16.2	Very low	24	31.5	31.3	32.6	37.5
**BAL**	511	14	Very low	22.4	35.2	35.6	36.0	0
**BAL**	443	18.8	Very low	23.1	33.8	32.7	33.2	38.3
**BAL**	482	14	Very low	21.7	31	30.6	30.9	36.4
**BAL**	377	16.1	Very low	24.5	33.6	33.5	34.9	0
**Sputum**	864	13.8	Very low	20.8	33.2	31.8	33.2	0
**BAL**	49	14.5	Very low	22.6	35.5	34.8	35.4	0
**BAL**	161	15.2	Very low	22.0	31.1	30.4	32.0	38.4
**BAL**	155	15.6	Very low	20.6	29.9	29.4	31.4	37.1
**BAL**	155	42	Very low	24.2	30.9	31.9	33.8	37.9
**BAL**	129	15.7	Very low	22.4	31.6	30.9	32.2	38.4
**Sputum**	409	16	Trace	26.1				
**BAL**	448	13.7	Trace	23.8				
**Sputum**	87	13.9	Trace	26.1				
**BAL**	575	13.9	Trace	24.7				
**BAL**	210	18.4	Trace	26.2				
**BAL**	281	21	Trace	24.6				
**Pleural fluid**	1319	10.6	Very low	26.3	33.2	33.0	34.7	39.1
**Lymphnode**	807	15	Very low	20.7	34.1	33.1	34.1	39.6
**Gastric aspirate**	680	14.8	Very low	21.4	33.6	32.1	33.3	37.9
**Biopsy**	526	25.8	Very low	23.8	34.1	34.0	35.0	39.5
**Lymphnode**	209	17.5	Very low	22.1	34.9	32.3	35.2	39.8
**Biopsy**	240	13.2	Very low	24.1	33.7	32.6	34.4	38.9
**Lymphnode**	259	18.8	Very low	23.7	35.8	35.4	35.7	0
**Biopsy**	147	16.5	Trace	26.4				
**Biopsy**	916	26.2	Trace	23.2				
**Biopsy**	478	18.6	Trace	25.0				
**Lymphnode**	526	13.7	Trace	25.0				
**Lymphnode**	562	17.7	Trace	24.7				
**Pleural fluid**	246	14.4	Trace	23.9				

^**#**^Time to positivity: number of days from time of MGIT inoculation to the positive culture result

* BAL: Bronchoalveolar lavage

All 68 isolates were phenotypically susceptible to Rifampicin; molecular Rifampicin susceptibility was determined in 19 of the 31 Ultra-positive samples with 100% concordance with phenotype. The results of the last 12 samples were “trace”, therefore Rifampicin susceptibility could not be assessed. The new category “trace” allowed 17.6% (12/68) of MTB Xpert-negative culture-positive samples to be detected.

## Discussion

Ultra was developed as a new generation assay to overcome Xpert’s limitations, particularly in smear-negative TB samples. We performed a head-to-head comparison between Ultra and Xpert on respiratory and non-respiratory samples previously found Xpert-negative but MTB culture-positive.

In our previous study on 234 smear-negative culture-positive TB samples (137 respiratory and 97 non-respiratory), the older version of Xpert found 171 samples positive and 63 negative with an overall sensitivity compared to culture of 73.1% (171/234): 73.0% (100/137) for pulmonary TB and 73.2% (71/97) for extra-pulmonary TB, respectively [9]. In the present study Ultra improved MTB detection by 45.6% compared to Xpert in smear-negative culture-positive TB samples. Therefore, we can speculate that Ultra could have detected a further 29 samples in our previous work (45.6% of the 63 Xpert-negative samples), increasing the overall sensitivity on smear-negative samples from 73.1% for Xpert to 85.4% for Ultra (171+29 = 200/234). A previous comparative study on Xpert and Ultra assays on 109 smear-negative sputum samples showed sensitivities of 66.1% and 78.9% respectively [6]. In the multicentre study designed by FIND, involving 1520 adults suspected of pulmonary TB in settings of varying prevalence of TB/HIV and MDR-TB, the direct head-to-head comparison on sputum samples revealed that the overall sensitivity of Ultra was 5% higher than that of Xpert compared to culture (87.8% vs. 82.9%), in particular Ultra sensitivity in 259 smear-negative patients was 73.4% [7]. In a recent multicentre accuracy study involving 2120 patients, an increase in sensitivity of 17% (from 46% to 63%) was observed in 137 subjects with smear-negative culture-positive sputum analyzed with both Xpert and Ultra [8].

We found that the mean time to positivity of samples found negative by Ultra was significantly longer in liquid culture, in comparison to positive samples, as previously shown by Rufai et al using Xpert assay [11].

Despite long term storage at -20°C, the detection rate by Ultra remained high; in contrast with Singh et al. who analysed gastric aspirates and induced sputum samples with Xpert. In our study the detection rate of the Ultra assay did not decrease significantly with increasing duration of storage [12].

No correlation between *rpoB* and IS1081/IS6110 probes Ct values and TTP or time of storage was observed, due to the very low semi-quantitative mycobacterial load of our samples.

Our study has several limitations: i) small sample size, since it was a monocentric study on selected frozen samples; ii) lack of negative controls as the aim of our study was a comparative analysis of previously collected MTB-positive Xpert-negative samples and not a specificity assessment; iii) Xpert-positive samples were not included as we expected them to be Ultra-positive.

In conclusion, our results are consistent with the few studies published so far and confirm the better performance of Ultra compared to the previous version in both respiratory and non-respiratory smear-negative samples, with an overall improvement of 45.6%.
